# A Wearable Internet of Things-Based Device for the Quantitative Assessment of Hand Tremors in Parkinson’s Disease: The ELENA Project

**DOI:** 10.3390/s25092763

**Published:** 2025-04-27

**Authors:** Yessica Saez, Cristian Ureña, Julia Valenzuela, Antony García, Edwin Collado

**Affiliations:** 1 Universidad Tecnológica de Panamá, Panama City 0819-07289, Panama; yessica.saez@utp.ac.pa (Y.S.); cristian.urena@utp.ac.pa (C.U.); antony.garcia@utp.ac.pa (A.G.); 2 Centro de Estudios Multidisciplinarios en Ciencias, Ingeniería y Tecnología AIP (CEMCIT AIP), Panama City 0819-07289, Panama; 3 Ministerio de Salud, Hospital Regional Anita Moreno, La Villa de Los Santos 0819-11380, Panama; ferisa2528@gmail.com

**Keywords:** inertial sensing, internet of things, tremor analysis, tremor monitoring, Parkinson’s disease

## Abstract

Parkinson’s disease (PD) is a progressive neurodegenerative disorder characterized by motor symptoms, with tremors being one of the most prominent. Traditional assessment methods, such as the Unified Parkinson’s Disease Rating Scale (UPDRS), rely on subjective, intermittent evaluations, which can miss symptom fluctuations. This study presents the development and validation of the ELENA system, an IoT-based wearable device designed for the continuous monitoring of tremors in PD patients and medication tracking in PD patients. Named in honor of a 67-year-old woman who has lived with Parkinson’s since 2011 and inspired the project, the ELENA system integrates an MPU6050 accelerometer, an ESP32 microcontroller, and cloud-based data analysis and MATLAB. The ELENA system was calibrated and validated against an Apple Watch, demonstrating high accuracy with frequency deviations under 0.5% and an average percentage error of −0.37%. Unlike commercial devices, ELENA offers a clinical-grade solution with customizable data access and visualization tailored for healthcare providers. Participants, including PD patients and a non-PD control group, completed a series of clinical tasks to evaluate tremor monitoring capabilities. The results showed that the system effectively captured tremor frequency and amplitude, enabling the analysis of resting, action, and postural tremors. This study highlights the ELENA system’s potential to enhance PD management by providing real-time, remote monitoring of tremors. The scalable, cost-effective solution supports healthcare professionals in tracking disease progression and optimizing treatment plans, paving the way for improved patient outcomes.

## 1. Introduction

Parkinson’s disease (PD) is a progressive neurodegenerative disorder characterized by motor symptoms such as tremors, bradykinesia, rigidity, and postural instability. Among these, tremors are one of the most prominent symptoms, and resting tremors are often the first noticeable sign for many patients [1].

A tremor is defined as a rhythmic, involuntary oscillatory movement of a body part [2]. In Parkinson’s disease (PD), tremors are commonly observed at rest, characterized by involuntary rhythmic shaking when the limb is supported and at rest.

Approximately 75% of people with PD experience tremors at rest at some stage of the disease [3,4,5,6,7], while nearly 60% develop tremors during voluntary movement [8,9,10]. Although the tremors subside with movement, they return when the limb is at rest, affecting motor control and affecting daily activities. Furthermore, around 25% of patients with PD also exhibit action tremors, further affecting functional ability [8]. Beyond tremors, PD is also linked to postural instability, swallowing difficulty, and a characteristic stooped posture, all of which contribute to a gradual decline in mobility and independence.

Traditionally, the clinical assessment of the symptoms of PD has relied on subjective evaluation methods such as the Unified Parkinson’s Disease Rating Scale (UPDRS) [11,12]. Although UPDRS remains the standard for clinical evaluation, its reliance on intermittent in-person evaluations introduces limitations. Current treatment relies primarily on medications, but they often provide limited relief from symptoms and can cause side effects that can be as challenging as the tremor itself.

Several pharmacological treatments exist for the treatment of tremors, including beta blockers (e.g., propranolol) and dopaminergic agents (e.g., levodopa for Parkinsonian tremors) [1,13,14]. However, these medications often provide limited relief from symptoms [15] and are associated with significant side effects, such as fatigue, dizziness, nausea, orthostatic hypotension, dyskinesia, hallucinations, and more [13,16].

In cases where pharmacological management is ineffective, surgical interventions such as deep brain stimulation (DBS) may be considered [17]. Although DBS is an effective therapy to reduce the severity of tremors, it is an invasive and high-cost procedure with potential complications, such as cognitive side effects [18].

Given these challenges, there is a growing need for alternative, non-invasive, and more effective approaches to tremor management to improve patient quality of life.

With the advancement of wearable and Internet of Things (IoT) technologies, there has been a shift toward more objective continuous monitoring solutions for the management of PD [19]. IoT-based systems, when integrated with wearable devices, provide the opportunity to monitor PD symptoms in real time, offering a more complete picture of disease progression and patient response to treatment [20]. These technologies could transform PD treatment by enabling remote monitoring, reducing the need for frequent clinic visits, and allowing timely therapeutic adjustments based on continuous data [19,21,22].

Wearable technologies, such as inertial measurement units (IMUs), are particularly promising for the quantification of tremors and other motor symptoms in PD patients. IMUs, equipped with accelerometers and gyroscopes, allow for the precise capture of motion dynamics, offering a quantitative approach to measuring tremors [20]. This is a significant improvement over traditional methods, such as UPDRS, which are highly dependent on human observation and can miss subtle changes in the severity of symptoms [23]. Thus, quantitative tremor measurements can provide clinicians with objective data, which can lead to more accurate diagnoses and more personalized treatment plans.

Recent research demonstrates the clinical utility of these quantitative measures. For example, a study by Delrobaei et al. (2018) showed significant correlations between tremor severity scores obtained from wearable sensors and UPDRS scores, suggesting that IoT-based systems could provide a reliable alternative for the evaluation of PD [20]. These systems also enable the monitoring of tremors in various body parts, offering a full-body assessment that is difficult to achieve with traditional clinical methods. By capturing tremor dynamics in real time, wearable devices can support clinicians in tracking symptom fluctuations and optimizing treatment regimens, especially for patients undergoing therapies such as DBS.

Recent research demonstrates the clinical utility of continuous, quantitative tremor measurements. Delrobaei et al. (2018) showed significant correlations between tremor severity scores obtained from wearable sensors and traditional UPDRS scores, suggesting that IoT-based systems could provide a reliable alternative for evaluating Parkinson’s disease (PD) [20]. Similarly, Espay et al. (2019) highlight that continuous monitoring technologies significantly improve the detection of subtle symptom fluctuations not captured by intermittent clinical assessments, facilitating more timely and personalized interventions [24]. Additionally, Lipsmeier et al. (2018) provided evidence that continuous digital measurements collected through wearable and smartphone sensors yield reliable, valid, and clinically meaningful data, enhancing clinicians’ understanding of symptom variability and progression [25]. These systems also enable comprehensive tremor monitoring in multiple body parts, offering full-body assessments that are difficult to achieve with traditional methods. By capturing tremor dynamics in real time, wearable devices support clinicians in tracking fluctuations in symptoms and optimizing treatment regimens, particularly for patients undergoing therapies such as deep brain stimulation (DBS). However, successful clinical integration of such detailed measurements necessitates proper clinician training and intuitive data visualization tools.

The use of accelerometers, a key component of these wearable systems, has become increasingly significant in both research and clinical settings. According to both reviews, Peña et al. (2024) and Mughal et al. (2022), accelerometers offer precise, real-time tracking of movement, which is crucial to monitoring motor symptoms of PD, such as tremors, bradykinesia, and rigidity [26,27]. Adams et al. (2017) [28] conducted a pilot study evaluating the feasibility of multisensor wearable technology for objective movement assessment in patients with Parkinson’s disease and Huntington’s disease. The study used accelerometer-based sensors at multiple body locations to track patient activities in both the clinic and at home for a 48 h period. The results showed that people with PD exhibited altered activity patterns, spent more time sedentary, and underwent fewer state transitions than healthy controls. Moreover, the use of wearable sensors provided objective continuous insight into motor symptoms that were not captured during episodic clinical evaluations. Later, in 2021, Adams et al. [6] conducted a real-world study using wearable sensors to assess motor characteristics such as tremors, gait, and activity levels in patients with PD over a two-day period. Their study found that the tremor frequency ranged between 3 and 10 Hz, with significant variation depending on the activity state. In particular, PD patients exhibited tremors for a median of 1.6 hours per day in their most affected hands, which was significantly higher than in less affected hands or control subjects. Additionally, these studies have shown that accelerometers, when integrated into IoT systems, not only provide continuous data streams, but also allow remote patient monitoring. This improves the frequency and precision of symptom tracking compared to traditional in-clinic evaluations.

Moreover, research emphasizes the versatility of accelerometers in distinguishing between different types of tremor, such as rest vs. action tremors [23,29,30,31]. These sensors can capture subtle variations in the frequency and amplitude of the tremor that are often missed during episodic clinical assessments. This capability makes them a powerful tool for both early diagnosis and ongoing monitoring, helping clinicians to tailor treatment strategies more effectively [32,33].

Further advances in the use of wearable accelerometers have also included integration with machine learning (ML) models [34,35]. These models analyze data to predict disease progression or therapy outcomes, offering a new frontier in personalized medicine. Therefore, continuous data collected through accelerometers are vital for refining these models and ensuring that motor and nonmotor symptoms are adequately monitored.

These technological advances in wearable accelerometers, coupled with the integration of ML models and IoT platforms, have opened new possibilities to improve the continuous monitoring and management of Parkinson’s disease symptoms. In this context, the ELENA project aims to build on these innovations by developing a low-cost, IoT-based wearable system specifically designed for monitoring PD tremors. The system leverages an IMU sensor for real-time data collection, which is transmitted over the Internet to IoT platforms for remote monitoring by healthcare providers. This approach enables clinicians to continuously observe tremor patterns and intervene as necessary, improving patient outcomes [36].

In this paper, the development and validation of the ELENA wearable system is presented, focusing on its hardware components, calibration processes, and the use of advanced algorithms for tremor frequency analysis. Using IoT technology and wearable devices, the ELENA Project provides a scalable and accessible solution to continuous tremor monitoring [36]. The project is named ELENA in honor of a 67-year-old woman who has lived with Parkinson’s disease since 2011 and inspired the motivation behind this research. This system builds on these technological advances by integrating real-time tremor monitoring with a medication management subsystem in a single wearable platform. Its low-cost, open-source design, and IoT-based architecture offer an accessible solution that enables cloud-based data visualization and remote clinical support. In contrast to commercial devices, which are not optimized for clinical tremor analysis or medication adherence, ELENA is specifically designed to support the longitudinal monitoring and therapeutic management of PD.

### The ELENA Project: Overview of Functionality

The ELENA Project is an IoT-based system designed to provide continuous monitoring of tremors in patients with PD while also addressing medication management needs [36]. The system aims to overcome the limitations of traditional intermittent clinical assessments by enabling remote real-time observation of motor symptoms, particularly hand tremors. It integrates wearable technology, cloud-based data processing, and a user-friendly interface for healthcare providers, caregivers, and patients to improve management and outcomes.

**Figure 1:**
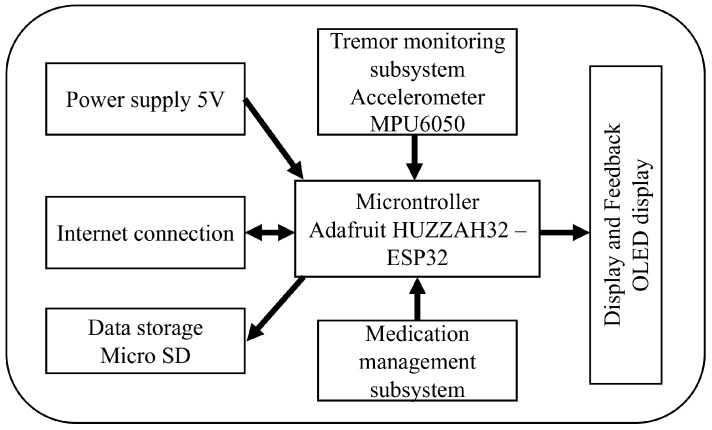
Conceptual diagram of the ELENA wrist-worn wearable prototype.

**Figure 2:**
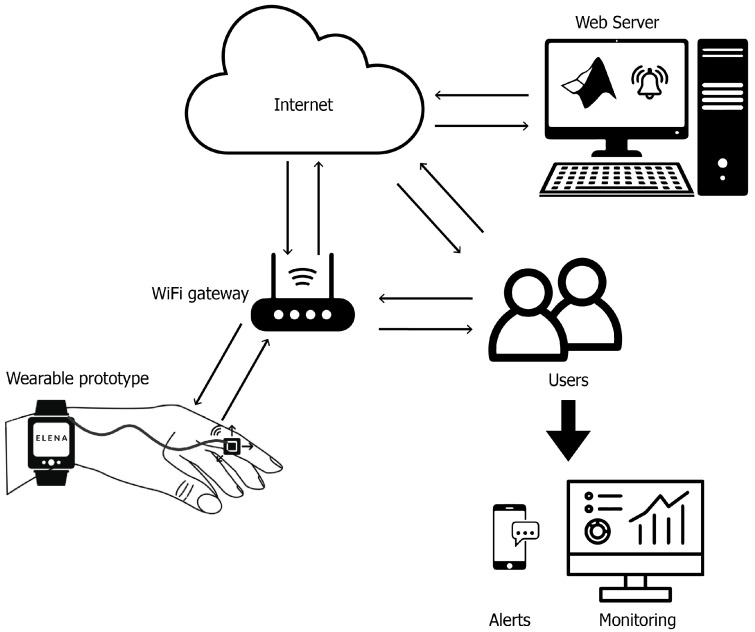
Functionality of ELENA Project.

The system consists of two subsystems: the medication management subsystem and the tremor monitoring subsystem (see Figure 1). These subsystems work together to provide comprehensive monitoring of upper limb movements and medication reminders, while facilitating communication between patients, caregivers, and healthcare professionals, as shown in Figure 2.

The medication management subsystem of the ELENA Project helps PD patients adhere to their treatment regimen while tracking the effectiveness of the medications. It provides automated reminders through a mobile app or wearable notifications, ensuring timely medication intake. Patients can log doses, and the system tracks adherence and missed medications, alerting caregivers if necessary. By correlating tremor data with medication intake, the system enables the real-time assessment of treatment efficacy, helping healthcare providers to optimize therapy. Currently, medical personnel can remotely review and interpret the stored data, providing personalized adjustments and proactive clinical interventions. Future developments aim to integrate artificial intelligence algorithms to automate and optimize this analytical process further. All data are stored in the cloud for remote access, supporting personalized adjustments and proactive interventions.

Although the medication management subsystem plays an important role in improving adherence and treatment effectiveness, it is not the primary focus of this work. Instead, this work mainly focuses on the tremor monitoring subsystem and its role in continuous real-time assessment of motor symptoms in patients with PD.

At the core of the tremor monitoring subsystem is a wearable device equipped with an IMU, specifically an MPU6050 accelerometer, which continuously captures motion data. The sensor is configured to monitor upper limb tremors by detecting changes in frequency and amplitude. This real-time data collection allows the system to track tremor patterns as patients perform daily activities, giving information to healthcare providers to observe trends and intervene as necessary. The data are transmitted to the cloud via Wi-Fi using an ESP32 microcontroller, where they are stored and analyzed on an IoT platform. Through this platform, healthcare providers can access the data remotely, gaining insight into symptom progression, and enabling timely interventions. Advanced signal processing techniques, including the FFT method, are employed for frequency analysis to identify dominant tremor frequencies and amplitudes.

Ultimately, the ELENA Project is designed and built to provide a scalable and accessible solution for continuous tremor monitoring and medication management, addressing both motor symptom tracking and adherence to treatment for PD patients. Using IoT technology and wearable devices, the system improves patient care through real-time data collection and remote monitoring, offering a new avenue to optimize PD treatment.

## 2. Materials and Methods

### 2.1. Materials

This study was conducted as part of the ELENA Project, an IoT-based initiative to help people with PD. The tremor monitoring system includes a wearable prototype, specifically designed for continuous tremor monitoring and medication adherence. The prototype is composed of the following components:

- **Microcontroller:** The system uses an Adafruit HUZZAH32—ESP32 Feather Board, which integrates Wi-Fi connectivity and provides sufficient processing power to handle data collection and transmission.
- **Accelerometer:** The MPU6050, a 6-degree-of-freedom (DoF) sensor that combines a 3-axis accelerometer and a 3-axis gyroscope, is used for precise tracking of upper limb movements.
- **Data Storage:** A micro SD card module is included for local storage of the tremor data collected, ensuring data retention even in case of connectivity issues.
- **Display and Feedback:** An OLED display (0.96” I2C 128 × 64 SSD1306) is used to provide real-time information to the patient. In addition, an auditory buzzer is integrated to issue alerts for medication reminders.
- **Power Supply:** The prototype is powered by a 3.7 V 2000 mAh lithium-ion battery, rechargeable via a USB connection for continuous operation.

The following software and equipment were used in the design, programming, and calibration of the wearable system.

- **Software for Programming the Wearable:** The microcontroller was programmed using the Arduino IDE (Integrated Development Environment) 2.0.3, which supports C++ programming and allows easy integration of the accelerometer through libraries such as Adafruit’s MPU6050 and Wire.h for I2C communication. This setup facilitated data acquisition, storage, and communication to the cloud platform.
- **Software for Constructing the Prototype:** The physical design of the wearable device was created using CAD software (Tinkercad 2.0.1) for the initial prototyping and Fusion 360 for the final design. The prototype casing was 3D printed using PLA (polylactic acid) material, providing a lightweight and durable enclosure for the electronics.
- **Calibration Equipment:**
  – **Signal Generator and Vibration Generator:** A combination of a signal generator (Siglent Technologies SDG2042X Arbitrary Waveform Function-Generator, 40 MHz) and a vibration generator was used to produce controlled sinusoidal signals across a range of frequencies. This setup allowed for the generation of known frequencies, which were essential for verifying the accuracy of the accelerometer’s measurements and for testing the system’s response to specific frequency inputs.
  – **Calibration Table:** The calibration process used a MTS uniaxial vibration table (manufactured by Material Testing Systems, MTS), which has a platform size of 1.5 cm and moves in one direction (usually vertical); this is commonly used for seismic simulation. The table allowed for precise control over low-frequency and low-amplitude vibrations, which was necessary for testing the sensor’s response to controlled oscillations. This table was equipped with software to program specific frequencies and amplitudes, ensuring accurate calibration of the MPU6050 sensor.
  – **Reference Watch:** Apple Watch Series 9 (S9 41 SI AL SB SB SM GPS-CLA, 41 mm, Silver Aluminum case, Sport Band, GPS model) equipped with the Vibration Analysis application was used to validate and calibrate the prototype. The Apple Watch Series 9 contains built-in accelerometers capable of capturing motion data at sampling rates up to 100 Hz. The Vibration Analysis app leverages this capability to measure tremor frequency and amplitude accurately, providing a reliable benchmark for comparison with the developed wearable system.

### 2.2. Tremor Analysis Software

Tremor analysis was performed using MATLAB, a versatile software environment suitable for handling time-series data and performing frequency analysis. MATLAB was used to process the accelerometer data collected from the wearable device, performing a detailed analysis of the tremor patterns. The following steps were implemented using custom MATLAB scripts:

- Data Preprocessing: The raw accelerometer data were imported into MATLAB, where it was filtered using a Butterworth low-pass filter to remove high-frequency noise and improve the accuracy of tremor detection.
- Frequency analysis: Fast Fourier transform was applied to transform the time-domain data into the frequency domain, enabling the identification of the dominant tremor frequencies. The analysis focused on extracting tremor frequencies in the typical ranges of Parkinson’s disease (4–6 Hz for resting tremors).
- Data Visualization: MATLAB’s plotting functions were used to create visual representations of the tremor data, including time-series plots and power spectral density (PSD) graphs. These visualizations were uploaded to ThingSpeak for seamless data processing, real-time visualization, and improved tremor analysis, making it easier for healthcare providers to track and manage the progression of Parkinson’s disease symptoms in real time.

These materials and tools enabled the development and precise calibration of the wearable system, ensuring its accuracy and reliability to monitor motor symptoms in patients with PD.

The wearable prototype was designed to be worn on the patient’s wrist, with the accelerometer positioned on the index finger. This setup guarantees the measurement of tremors in both resting and active conditions, providing detailed data for analysis.

The designed wearable prototype facilitates the monitoring of tremors using three different methods. First, data can be stored on a microSD module for offline analysis. Second, the system can measure the accelerations of the tremors and transmit the data to an IoT platform, where it is stored in a searchable database that allows access and downloads as needed. Lastly, the wearable includes a direct wired connection for real-time monitoring, which is particularly useful for clinical evaluations during hospital visits.

### 2.3. Participants

The study included two groups: a PD group, recruited from the Anita Moreno Regional Hospital in Los Santos Province, Panama; and a non-PD control group recruited from Los Santos Province, Panama.

- **PD Group:** This group consisted of 9 patients diagnosed with Parkinson’s disease by certified neurologists. The inclusion criteria for the PD group were as follows:
  – A confirmed diagnosis of Parkinson’s disease according to the clinical diagnostic criteria of the Brain Bank of the UK Parkinson’s Disease Society.
  – Age between 60 and 80 years of age.
  – Ability to provide informed consent and complete the study protocol.
  – Stable medication regimen for at least 30 days prior to the study.
  – No history of other neurological or musculoskeletal disorders that could interfere with motor assessments.
- **Control Group:** The control group included 9 participants of the same age without neurological conditions. The inclusion criteria for the control group were as follows:
  – Age between 60 and 80 years of age, matched as closely as possible to the PD group to control for age-related variability.
  – No history of neurological or musculoskeletal disorders.
  – No regular use of medications that affect the central nervous system.
  – Ability to provide informed consent and complete the study protocol.

Both groups underwent similar assessments using the IoT-based tremor monitoring system. The demographic details of the PD participants are shown in Table 1, while the demographic details of the control group are shown in Table 2. All participants, both those with PD and those in the control group, provided their written informed consent before participating in the study. The study protocol was approved by the Panama National Research Bioethics Committee (Comité Nacional de Bioética de la Investigación de Panamá, CNBI), under the internal number EC-CNBI-2023-02-176. The approval was granted on 11 July 2023 for the protocol titled “IoT-based assistive technology for monitoring motor symptoms in individuals with Parkinson’s disease (Proyecto Elena)”.

### 2.4. Methods

#### 2.4.1. Assessment Tools

**Figure 3:**
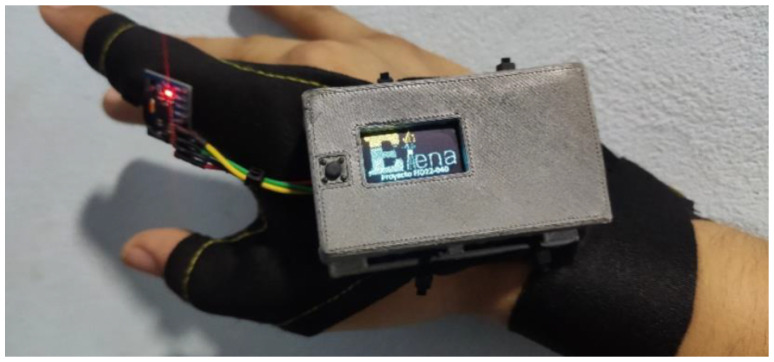
ELENA wrist-worn wearable prototype.

The assessment of tremor monitoring was performed using a custom-built IoT-based device developed as part of the ELENA Project. This subsystem system consisted of two primary components:

- **Wrist-worn wearable prototype:** This device consists of a microcontroller (Adafruit HUZZAH32—ESP32 Feather Board) housed inside a 3D-printed case and an accelerometer (MPU6050) positioned on the index finger to measure tremors in the upper extremities (see Figure 3). In addition, a glove was designed to securely hold the prototype, ensuring proper sensor placement and stability during use. The glove used to hold the wearable device was specifically designed to be lightweight, comfortable, and non-restrictive, ensuring minimal interference with natural tremor movements. Preliminary tests indicated no observable worsening or improvement of tremors due to the glove’s presence. This was confirmed through direct patient feedback and clinical observation during validation sessions.
- **Data collection platform:** The device was configured to store the collected data on a micro SD card and also transmit it to a cloud-based platform for visualization and analysis using MATLAB software. The monitoring system also provided real-time feedback to patients and their caregivers through an integrated OLED display and audible alerts.

#### 2.4.2. Calibration Tests

**Figure 4:**
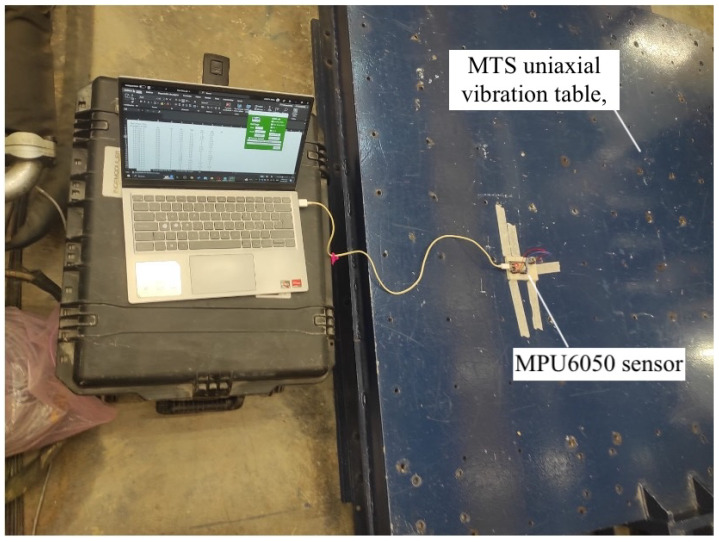
MPU6050 sensor calibration.

The calibration of the monitoring system was essential to ensure an accurate measurement of the frequency and amplitude of the tremor. The following calibration procedures were performed:

1. **Sensor Calibration:** This calibration was conducted using a six-position method on a calibration table, as seen in Figure 4.

The MPU6050 sensor was mounted on a cube so it could be placed in six different static orientations. Each axis of the accelerometer was aligned parallel to the force of gravity in turn, verifying that it measured approximately 1 g. The table setup allowed the consistent and reliable positioning of the sensor to ensure accurate calibration. For further accuracy and testing, a uniaxial vibration table from MTS was used for more advanced calibration. The vibration table had a platform size of 1.5 cm and was designed for seismic simulation, which allowed for precise control over frequencies and amplitudes. This table provided a controlled environment to simulate low-frequency and low-amplitude movements that are similar to the conditions experienced during tremor symptoms. The frequencies and amplitudes were programmed using the MTS software provided with the table. This setup allowed us to test the sensor’s response to known vibrations, ensuring that it was calibrated correctly across all axes.

**Figure 5:**
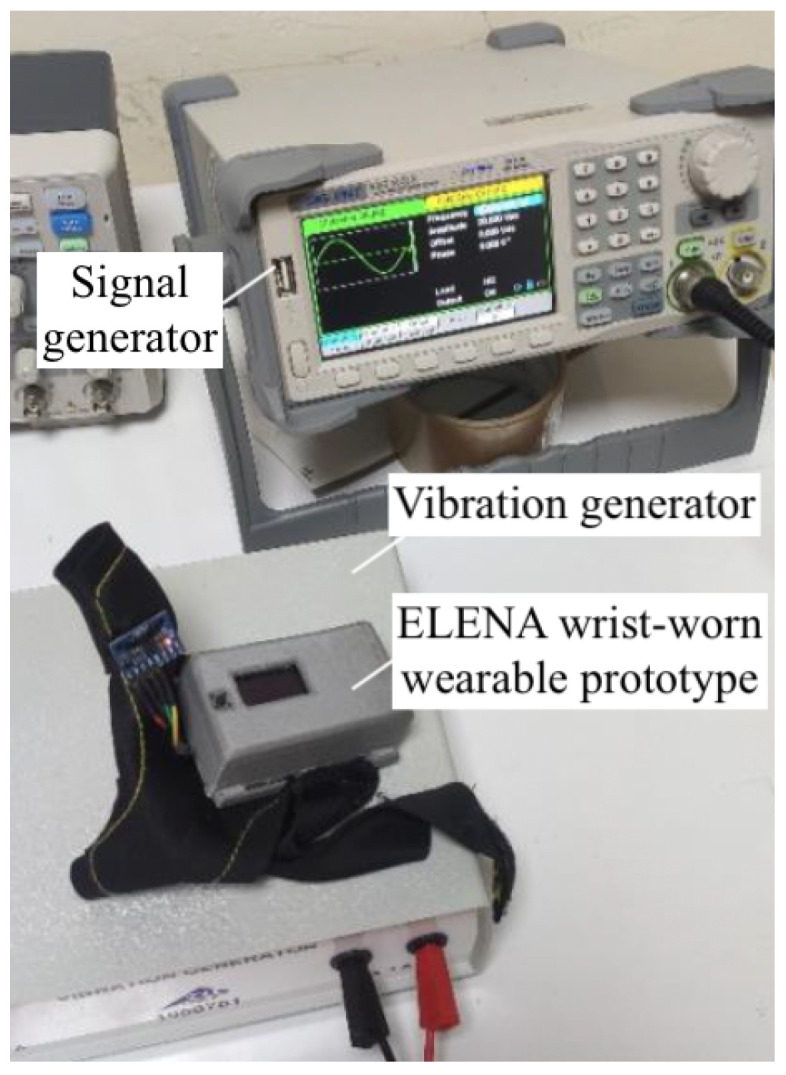
ELENA prototype setup on vibration generator for tremor simulation.

**Figure 6:**
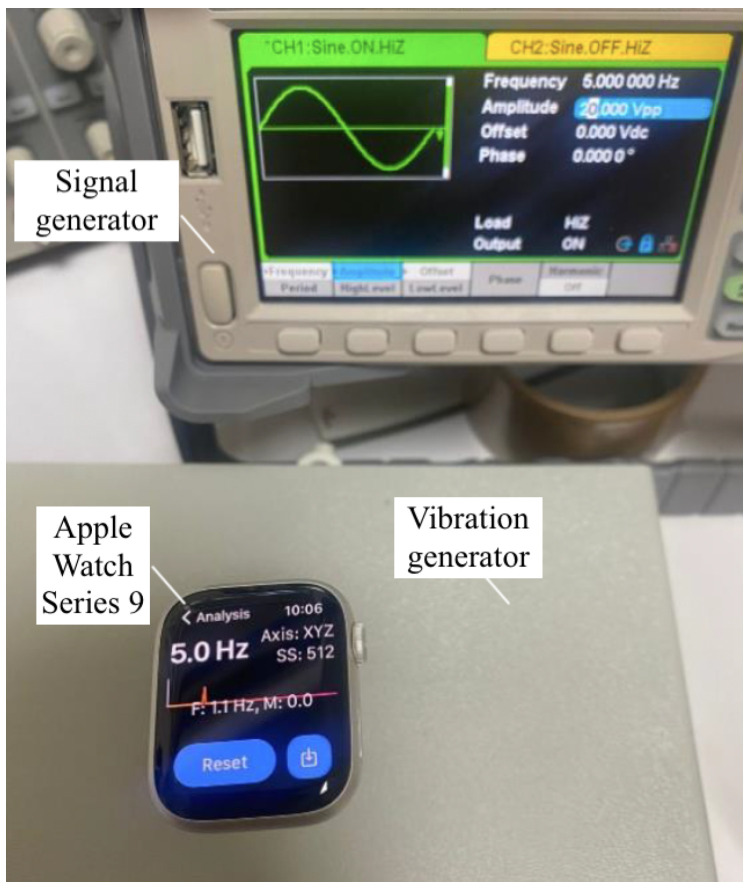
Apple Watch recording simulated tremor vibrations for prototype validation comparison.

2. **Signal Filtering:** A Butterworth low-pass filter was applied in MATLAB to reduce noise from the accelerometer data, achieving a more accurate detection of tremor frequencies.
3. **Accuracy Validation of the Prototype:** To validate the accuracy of the prototype, a series of controlled laboratory tests were conducted comparing data collected from the MPU6050 sensor in the prototype to readings from an Apple Watch. The collected data, processed via MATLAB for frequency and amplitude analysis, were stored on the SD card and transmitted to the cloud. The validation process involved three main steps: preparation of equipment, data collection and analysis, and result comparison.
  - **Preparation of Equipment:** The prototype was mounted on a vibration generator set to specific frequencies. Figure 5 ilustrates this procedure. Simultaneously, the Apple Watch of Figure 6, equipped with an accelerometer and a custom application for frequency analysis, was used as a benchmark device.
  - **Data Collection and Analysis:** Both devices recorded vibration data across a range of frequencies. Data from the prototype were processed using MATLAB to analyze frequency and amplitude. Results were stored locally on an SD card and transmitted to the cloud for further processing.
  - **Comparison of Results:** The analysis focused on the frequency values obtained from both devices. The key tests that were performed included the following:
    – **Static Frequency Test:** The frequencies were fixed and the results were compared for each frequency.
    – **Dynamic Frequency Test:** The vibration generator was set to vary the frequencies continuously.
    – **Error Analysis:** Repeated measurements were taken to calculate the percentage error for the prototype.

#### 2.4.3. Clinical Tasks and Procedure

Each participant completed a series of clinical tasks designed to simulate typical daily activities while wearing the IoT-based monitoring device:

- **Resting State Measurements:** Each participant completed four resting tests for each hand:
  1. Sitting with the hand and forearm resting on the legs for 1 min.
  2. Sitting with the upper extremity extended without support for 1 min.
  3. Standing with the hand and forearm resting on the legs for 1 min.
  4. Standing with the upper extremity extended without support for 1 min.
- **Active Movement Tests:** Participants performed two active tasks with each hand:
  1. Writing their name for 1 min.
  2. Simulating a waving gesture for 1 min.

A neurologist from the Anita Moreno Regional Hospital recommended this protocol, based on the studies normally conducted on patients.

#### 2.4.4. Tremor Frequency Calculation

The tremor frequency of each patient was calculated using data collected from the accelerometer. The following method was used:

1. **Data Collection:** Acceleration data along the X, Y, and Z axes were recorded at a sampling rate of 55.56 Hz for multiple test sessions per patient. The chosen sampling frequency was based on the typical frequency range of Parkinsonian tremors, which generally fall between 3 and 6 Hz, with some components potentially as low as 1 Hz and as high as 12 Hz. According to the Nyquist criterion, a minimum sampling rate of at least twice the maximum expected frequency is required to accurately capture the signal without aliasing. Our selected sampling rate of 55.56 Hz satisfies this condition and provides sufficient resolution for accurate tremor frequency analysis while maintaining efficient data storage and power consumption on the wearable system.
2. **Preprocessing:** The recorded data were processed to correct missing values, remove trends, apply a filter to eliminate low-frequency noise, normalize the signals, and convert acceleration values to standard units. Low-frequency components often arise from postural shifts, baseline drift due to sensor movement, or voluntary gross motor activity such as hand repositioning. For example, during tasks involving writing or arm extension, we observed slow baseline shifts and large-amplitude voluntary motions that produced dominant components below 1 Hz. These components could obscure the tremor-related frequency content and were effectively reduced using a high-pass Butterworth filter (cutoff frequency = 1 Hz), improving the accuracy of tremor frequency estimation.
3. **Frequency Analysis:** A Fast Fourier Transform was applied to the processed acceleration data to extract the dominant frequency components. The analysis considered the energy distribution across frequency bands to identify the most prominent frequency range of tremors. Since the MPU6050 sensor captures acceleration along three orthogonal axes (x, y, z), the power spectral density (PSD) was computed independently for each axis. The axis exhibiting the highest spectral peak was used to identify the dominant tremor frequency. Additionally, the total energy of the tremor signal was calculated by summing the squared acceleration values across all three axes within each window. This approach provides a more comprehensive measure of tremor intensity, accounting for multidirectional movement components and enhancing robustness against variability in axis orientation.
4. **Results Storage:** The extracted frequency components and amplitude values were stored locally and on the cloud for further statistical analysis.

## 3. Results

### 3.1. Results of the Calibration Tests

**Figure 7:**
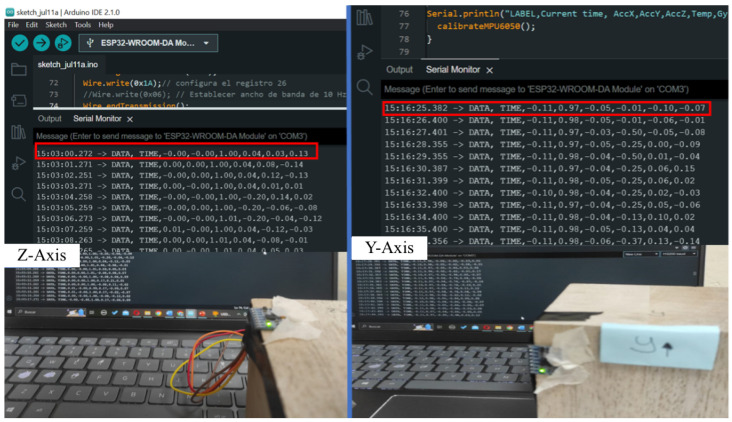
Calibration of the accelerometer along the z and y axes.

**Static Calibration:** The sensor was placed in six static orientations on a wooden cube. Each axis of the accelerometer was aligned parallel to the gravitational force, and the readings were analyzed to ensure that the sensor measured approximately 1 g (1 g $\approx 9.81\;m/s^{2}$) for each axis. Deviations were within ±0.02 g, indicating proper calibration for static conditions. Figure 7 illustrates the results for the z- and y-axes during static calibration, where the sensor was oriented to align the axes with the force of gravity. The acceleration values are the first three comma-separated numerical values (arranged as x, y, z) in each time-stamped data line shown in the data logger screen. For example, in the case of z-axis measurements, the third numerical value is the z-direction acceleration close to 1 g, whereas the x and y values are zero.

**Dynamic Calibration:** In addition to static testing, dynamic calibration was carried out using a uniaxial MTS vibration table. This setup allowed for the precise control (referring to the ability of the MTS vibration table to produce known, repeatable frequency and amplitude profiles with minimal deviation) of low-frequency (0.5 Hz to 10 Hz) and low-amplitude (0.1 g to 1 g) movements. The sensor response to these controlled vibrations was validated, with deviations in frequency measurements remaining below 0.5% and amplitude deviations below 2%. Dynamic tests also confirmed that the MPU6050 maintains stable and accurate readings, even under conditions that simulate tremor monitoring.

The successful integration of static and dynamic calibration processes ensures the reliability of the MPU6050 sensor for precise motion monitoring in real-world applications.

### 3.2. Results of the Accuracy Validations Tests

The accuracy validation tests compared the MPU6050 sensor’s performance to the Apple Watch under static and dynamic conditions, ensuring the reliability of the prototype for motion monitoring.

**Static Testing:** The prototype and the Apple Watch were tested at specific frequencies using a vibration generator. The accelerometer readings were closely matched, with deviations remaining minimal. However, the Apple Watch application, Tremor Analysis, did not measure or detect frequencies below 3 Hz, which limited comparisons at lower frequencies. Table 3 provides a detailed summary of key findings for both static and dynamic tests.

**Dynamic Testing:** The vibration generator was programmed to continuously vary frequencies, simulating real-world motion conditions. The prototype’s measurements aligned closely with those of the Apple Watch for frequencies above 3 Hz.

**Error Analysis:** Repeated measurements across multiple frequencies showed consistent accuracy, validating the sensor calibration and reliability. The ELENA system achieved a mean frequency deviation below 0.5% and an average percentage error of −0.37%.

These validation results complement findings from the previous literature. For example, Delrobaei et al. (2018) reported strong correlations (r = 0.74–0.77) between tremor severity scores obtained from wearable sensors and the UPDRS clinical rating scale, highlighting the clinical utility of sensor-based monitoring [20]. While their study focused on clinical correlation rather than signal-level precision, our results offer a quantitative device-level validation. Furthermore, the Apple Watch’s lower detection threshold (3 Hz) limits its ability to detect slow-frequency tremors. ELENA was validated across a broader frequency range (1–12 Hz). This is a significant limitation in the context of Parkinson’s disease, where postural or voluntary tremors—especially under cognitive load—may occur in the 1–3 Hz range. ELENA’s ability to detect tremors below this threshold enables the capture of clinically meaningful motor fluctuations that may otherwise go unrecognized in commercial wearable platforms.

### 3.3. Results of the Clinical Tasks and Procedure

For visualization purposes, we present graphical comparisons of the tremor characteristics for Test 1 and Test 2 among all patients alongside the control subjects. A summary of the results for all tests and all patients is provided in a table, as presenting individual results for each test separately would require excessive space.

**Analysis of Tremor Frequency Spectrum for Test 1**

**Figure 8:**
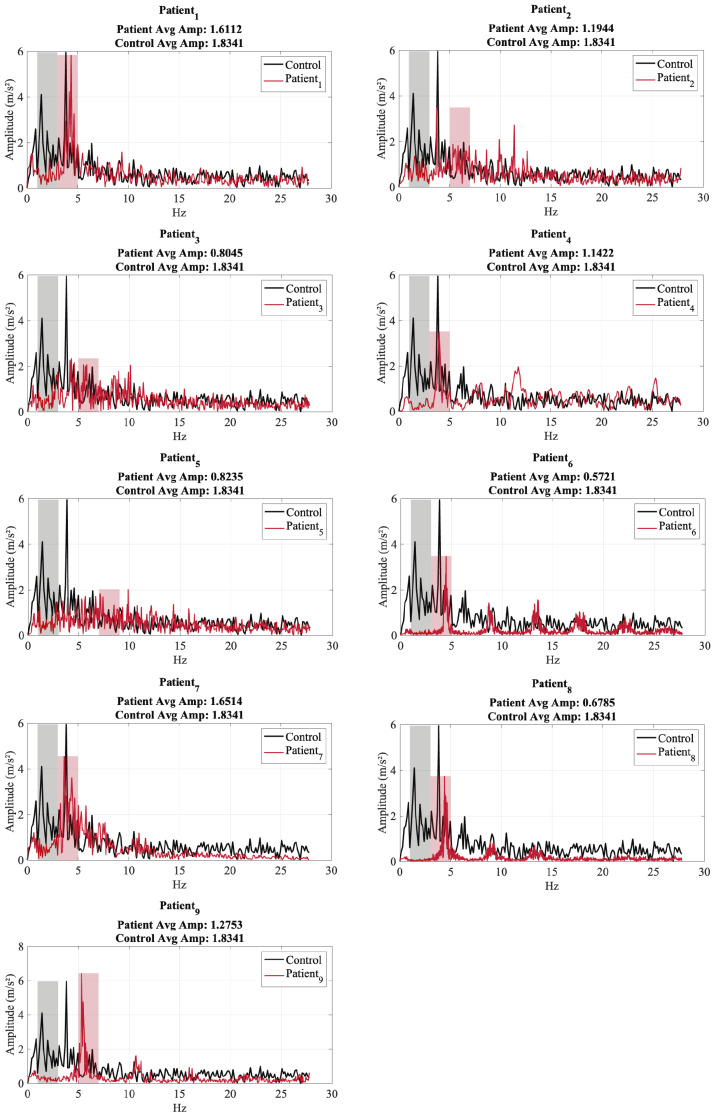
Tremor Frequency Spectrum for Test 1—patients (red) vs. control (black).

Figure 8 presents the tremor frequency spectra of nine patients (red) and a control subject (black) in resting condition, where the hand and forearm were placed on the legs. Each subplot compares the patient’s tremor spectrum with the control and highlights the dominant frequency zones: the control’s dominant frequency range is colored black, while the patient’s is colored red. The average amplitude within the dominant frequency zone is also displayed for both groups.

The control subject exhibits a dominant tremor frequency between approximately 3 and 6 Hz, which aligns with normal physiological tremors. In contrast, several patients display dominant frequencies within the 4–7 Hz range, which is characteristic of PD tremors. In addition, some patients show secondary peaks at higher frequencies (above 10 Hz), suggesting additional motor irregularities.

When comparing tremor amplitudes, the control subject generally has a higher average amplitude (1.83 m/s^2^) within its dominant frequency range. Although the tremor frequency ranges for PD patients and healthy individuals may appear to be similar, PD tremors distinctly differ in their rhythmicity, consistency, and persistence. These characteristics have been shown to play a more significant role in tremor classification than frequency alone, as highlighted in Dai et al. (2015) [37], where PD tremors exhibited stable dominant frequencies with sharp spectral peaks and consistent amplitude patterns across tasks [38]. Most patients exhibit lower amplitude values, but some (such as Patient_1_ and Patient_7_) show comparable or even higher amplitudes. This variation suggests differences in the severity of the tremor or the presence of other motion artifacts. Some patients, such as Patient_2_, Patient_3_, and Patient_6_, show lower tremor amplitudes at all frequencies, indicating milder tremors or different tremor characteristics. In contrast, patients such as Patient_1_ and Patient_7_ exhibit stronger tremors, with amplitudes approaching or exceeding that of the control subject. Patients with wider dominant frequency zones, such as Patient_4_ and Patient_9_, may have more complex tremor behaviors, possibly influenced by multiple sources.

**Analysis of Tremor Frequency Spectrum for Test 2**

**Figure 9:**
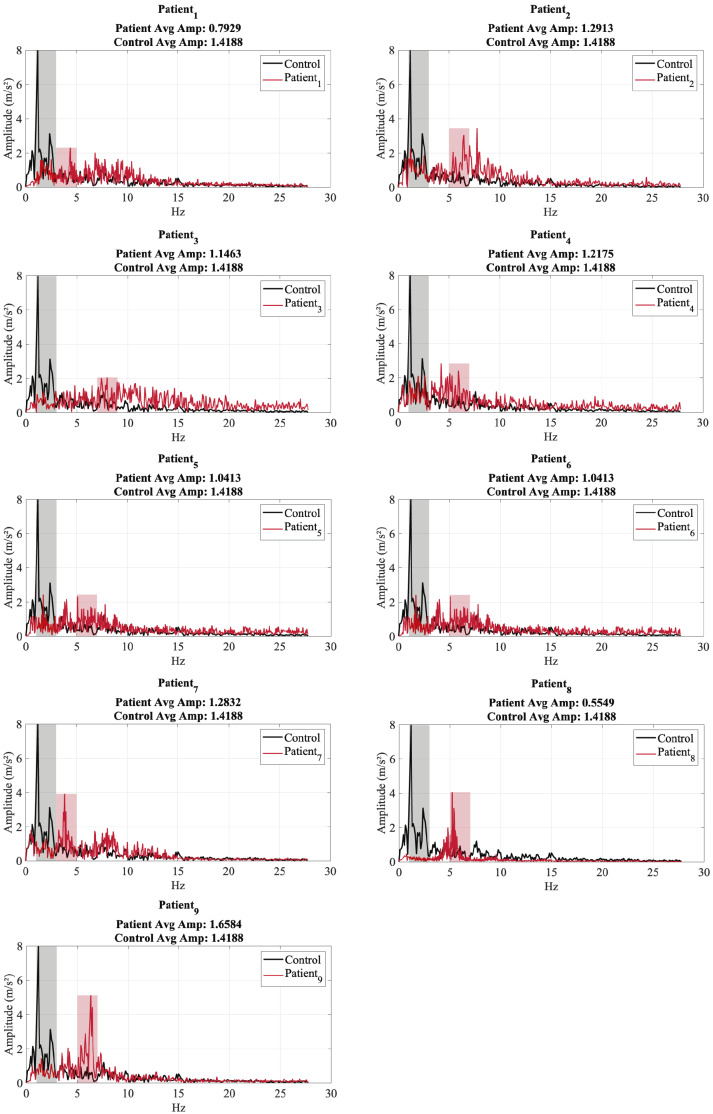
Tremor Frequency Spectrum for Test 2—patients (red) vs. control (black).

Figure 9 presents the tremor frequency spectra of the nine patients (red) and a control subject (black) under the conditions of Test 2. Each subplot compares the tremor spectrum of a patient with the control and highlights the dominant frequency zones for both: the control’s dominant frequency range is colored black, while the patient’s dominant frequency range is colored red. The average amplitude within the dominant frequency zone is displayed in each graph.

The control subject exhibits a dominant tremor frequency between approximately 3 to 6 Hz, which aligns with normal physiological tremors. Most patients show dominant frequency peaks in the 4–7 Hz range, which is characteristic of PD tremors. Several patients show additional peaks at higher frequencies (7–10 Hz and higher than 12 Hz), indicating possible secondary tremor sources.

When comparing amplitudes, the control subject’s average amplitude in the dominant frequency zone is lower in Test 2 (1.41 m/s^2^) than in Test 1 (1.83 m/s^2^), suggesting a possible decrease in background tremors. Several patients (such as Patient_2_, Patient_7_, and Patient_9_) exhibit higher average amplitudes, indicating stronger tremors under Test 2 conditions. Meanwhile, patients such as Patient_4_, Patient_6_, and Patient_8_ show lower tremor amplitudes, suggesting less pronounced tremors in this condition.

Some patients (like Patient_3_, Patient_6_, and Patient_8_) have reduced tremor amplitudes compared to Test 1, possibly indicating that the severity of the tremor varies based on the test condition. Others, such as Patient_2_, Patient_7_, and Patient_9_, exhibit increased tremor amplitudes, suggesting that certain conditions in Test 2 could exacerbate their tremors. Patients with broader dominant frequency zones (as Patient_7_ and Patient_9_) may have more complex tremor patterns, possibly influenced by multiple neurological or muscular factors.

A dominant peak within 4–7 Hz with increased amplitude suggests PD tremors. Patients whose dominant peaks overlap significantly with the control (3–6 Hz) may have milder tremors or early-stage Parkinson’s disease. The presence of secondary peaks above 7 Hz in some patients suggests additional neuromuscular dysfunctions that should be further analyzed.

When comparing Test 2 with Test 1, we observe that the amplitude of the control subject is lower in Test 2 than in Test 1, possibly due to changes in the conditions of the resting state. Some patients show an increase in the tremor amplitude in Test 2, while others show a decrease in tremor activity, suggesting task-dependent tremor behavior. Although most patients maintain their tremor frequencies in the 4–7 Hz range, secondary tremor peaks appear more frequently in Test 2, which may indicate motor activation related to tasks.

**Analysis of Parkinson’s Disease Tremor Data for All Patients and Tests**

The results of the tremor analysis for all tests and patients reveal key patterns in PD tremors. Patients at rest (**Test 1: sitting with the hand resting** and **Test 3: standing with the hand resting**) generally exhibit dominant frequencies in the **3–7 Hz** range, which corresponds well with the established resting tremor frequency of Parkinson’s disease (**3–8 Hz**). The energy concentration range in these resting conditions confirms that these tremors are effectively captured.

When patients extend their upper extremities without support (**Test 2** and **Test 4**), dominant tremor frequencies increase slightly, sometimes exceeding **7 Hz**. This suggests the presence of postural tremors, which typically have higher frequencies than resting tremors. In addition, the average acceleration amplitude increases under these conditions, indicating that removing postural support intensifies tremors.

The most significant variations appear in **Test 5** (writing for one minute) and **Test 6** (simulating a waving gesture). Some patients exhibit lower dominant frequencies (**1 Hz**), aligning with voluntary movement patterns, while others maintain tremor frequencies within the **3–8 Hz** range, suggesting that resting tremors persist even during movement. Also, tremor amplitude tends to increase during writing and waving tasks, implying that voluntary movement does not suppress tremors but, in some cases, may exacerbate them.

The presence of **NaN values** in certain tests indicates that some patients were unable to perform tasks such as writing or waving, which could be associated with disease progression and difficulty in voluntary movement. These findings reinforce clinical observations that motor symptoms, particularly fine motor control, are more affected in Parkinson’s disease. The results of the analysis of the frequency and amplitude of the tremor for all tests and patients are summarized in Table 4.

The intensity of the tremor varies significantly between different age groups in individuals with Parkinson’s disease. Understanding these variations can provide information on the progression of the disease and possible therapeutic interventions. This study examines the amplitude of the tremor in four age groups (60–64, 65–69, 70–74, and 75–79), showing differences in intensity and variability.

**Tremor intensity across different age groups**

**Figure 10:**
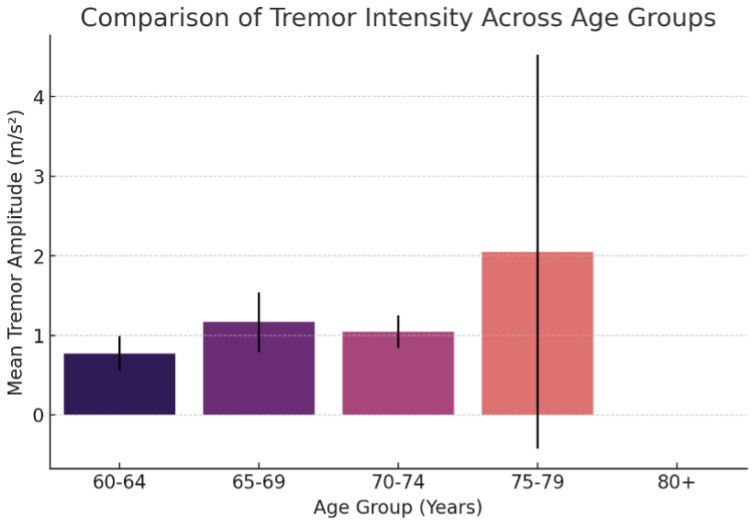
Comparison of tremor intensity across different age groups. The bars represent the mean tremor amplitude.

Figure 10 presents the comparison of the intensity of the tremor in different age groups, illustrating the mean tremor amplitude along with the standard deviation error bars. Furthermore, Table 5 summarizes the mean and variability of the intensity of the tremor for each age group.

We observe that patients in the 75–79 age group have the highest mean tremor amplitude (2.05 m/s^2^), accompanied by the greatest variability, as indicated by the large standard deviation. This suggests that for some individuals, the intensity of the tremor peaks in this age range, although considerable variability between patients is observed. In contrast, patients in the 65–69 and 70–74 age groups show moderate tremor amplitudes (1.16 m/s^2^ and 1.04 m/s^2^, respectively), pointing out that the intensity of the tremor increases relative to the younger age groups, but remains relatively stable before reaching the age category 75+. Patients in the 60–64 age group exhibit the lowest tremor intensity (0.77 m/s^2^) with reduced variability, which is consistent with the expected progression of Parkinson’s disease, where the severity of the tremors tends to worsen with age.

These findings show the progressive nature of Parkinson’s-related tremors and emphasize the importance of age-specific assessments in both clinical evaluations and treatment strategies. Further studies incorporating larger sample sizes and additional patient characteristics could provide more insight into the relationship between tremor intensity and disease progression.

## 4. Discussion

The analysis of tremor intensity across different test conditions and groups of patients provides valuable information on the nature and progression of PD tremors. Our findings indicate that resting tremors are consistently present, with dominant frequencies in the 3–7 Hz range observed in Tests 1 and 3, which aligns with the well-documented characteristics of Parkinson’s disease. In contrast, postural tremors, as seen in Tests 2 and 4, display higher frequency components exceeding 7 Hz, suggesting increased muscle activation and stabilization effort when the upper limb is not supported.

Motor tasks such as writing and waving (Tests 5 and 6) introduce greater variability in tremor frequencies, with some patients exhibiting very low-frequency tremors (<2 Hz). This probably reflects the contribution of voluntary motor control superimposed on pathological tremor activity. Moreover, the amplitude of tremors is notably highest in these motor tasks, supporting the hypothesis that voluntary movement exacerbates the intensity of tremors. The observation that some patients were unable to complete these fine motor tasks suggests that disease progression is correlated with greater difficulty performing precise movements.

A key implication of these results is that the tremor amplitude tends to increase with voluntary motor engagement, which may be influenced by motor and cognitive stress. This finding is consistent with previous research that indicates that cognitive load and emotional stress can modulate the severity of tremors. Furthermore, the variability in tremor intensity between patients, particularly in older age groups, suggests a differential progression of the disease, where some individuals experience an increased rigidity rather than an increased severity of tremors.

Among the recorded cases, one patient in the 75–79 age range showed the highest tremor amplitude. However, due to the small sample size and the presence of only one subject in this subgroup, this observation should be interpreted with caution and cannot be generalized. Younger patients (60–64 years) showed lower tremor amplitudes in this sample, which may reflect earlier disease stages, but further statistical validation is required.

Furthermore, examining the intensity of the tremor as a function of years since diagnosis suggests a progressive increase in the severity of the tremor over time. Patients diagnosed in the last 0–4 years exhibit the lowest mean tremor amplitude, whereas those who have been diagnosed for 5–8 years show a notable increase in intensity and variability. Tremor intensity remained elevated in patients diagnosed for 9–12 years, but high variability was observed. Due to the limited number of participants per group, no statistical inference can be drawn at this stage. These preliminary trends suggest the need for longitudinal studies and regression-based analysis with larger cohorts to more rigorously examine the relationship between disease duration and tremor characteristics.

## 5. Future Work

We acknowledge the importance of medical practitioners’ readiness for adopting new technologies. The successful clinical implementation of novel monitoring systems, such as ELENA, requires more than accurate and detailed data. Appropriate training programs for healthcare providers, user-friendly data visualization tools, and ongoing interdisciplinary collaboration between engineers, clinicians, and researchers are crucial to ensure that medical professionals can effectively interpret and utilize these new forms of quantitative tremor data. Future research should thus include validation studies and user interface design considerations in direct collaboration with medical practitioners to facilitate seamless integration into clinical practice.

Based on these findings, several key areas warrant further investigation. First, a comparative analysis of tremor intensity between different age groups should be conducted to explore potential age-related effects on tremor severity. Also, examining correlations between years since diagnosis and tremor characteristics will provide further information on disease progression, particularly in advanced stages (13 + years postdiagnosis) where data are currently limited.

Another important avenue of research is the differentiation between wrist and index finger tremors, if data are available separately for both. This distinction may provide a more granular understanding of motor control deficits and how they manifest in different limb segments. Exploring how cognitive load and emotional stress influence tremor intensity during testing conditions could also reveal key external modulating factors. In addition, future work will focus on the integration of all system components—including the tremor monitoring and medication management subsystems—into a cohesive platform. This will be accompanied by the development of a user-friendly interface, designed to support patients and healthcare providers with intuitive visualization tools, real-time feedback, and simplified interaction with the system.

Future studies should incorporate inferential statistical analysis, including p-values and confidence intervals, to strengthen the comparison between PD and control groups, as well as across age and diagnosis-year subgroups. Additionally, regression analysis will be applied to investigate correlations between tremor intensity and years since diagnosis. These analyses require larger and more balanced participant groups to ensure statistical power and reliability.

Future work will also include the implementation and validation of the ELENA system’s medication management subsystem and IoT capabilities. Although these features were part of the original system design, they were not the focus of this study. Planned functionalities include a mobile application for medication reminders, patient dose logging, and caregiver alerts. In addition, the system will be enhanced with cloud-based visualization tools to support clinicians in monitoring treatment adherence and assessing the relationship between tremor behavior and medication intake. These components aim to provide a more holistic and patient-centered tool for managing Parkinson’s disease.

As part of our future work, we aim to incorporate human activity recognition (HAR) capabilities into the system. This involves collecting movement data over extended periods while participants perform specific daily activities such as eating, brushing teeth or hair, waving, or writing. These data will be gathered under an approved bioethical protocol, which allows patients to use the wearable prototype in their homes after structured training sessions on proper usage and care. The resulting datasets will be used to develop machine learning models that can distinguish between different types of movements and their context, enabling the more precise interpretation of tremors and their impact on functional activities. This will enhance the clinical value of the system and allow for more personalized treatment recommendations.

Finally, addressing data collection gaps, such as expanding the sample size for patients 80 years and older, will enhance the generalization of the findings. Future studies should also consider incorporating objective motion capture and electromyographic (EMG) data to refine the characterization of tremor dynamics across different motor tasks. These efforts will contribute to a more comprehensive understanding of Parkinson’s tremors and help develop targeted therapeutic interventions.

### 5.1. Limitations

Although the ELENA system provides a comprehensive approach to tremor monitoring in PD, there are several limitations to consider. First, the cohort included only nine PD patients, which limits the statistical power of the findings and affects the robustness of inter-subject comparisons. This also impacts the generalizability of results to the broader PD population. The sample was limited to the patients available through our collaborating clinical partners, as recruiting individuals with Parkinson’s disease—particularly older adults or those with more advanced stages—is logistically and ethically challenging. Additionally, our study did not include participants over 80 years old or left-handed individuals, who may exhibit different tremor characteristics. Future validation studies will include a larger and more diverse participant pool across age groups, disease stages, and handedness to support broader clinical applicability. Furthermore, all participants were recruited from a single clinical network in Panama, which may introduce cultural and healthcare-related bias. Expanding future recruitment to multiethnic and geographically diverse populations will be important for improving external validity.

Second, the system focuses primarily on hand tremors and does not account for lower limb tremors or other motor symptoms such as bradykinesia and rigidity. Future work should expand the scope of movement analysis to provide a more complete assessment of motor impairments in PD.

Third, while wearable sensors effectively capture tremor frequency and amplitude, the study did not include long-term monitoring beyond test sessions. Continuous long-term data collection could provide deeper insight into tremor fluctuations throughout the day and in response to adherence to medication.

Fourth, the system relies on cloud-based data transmission, which may introduce latency and raises concerns regarding offline functionality and data availability in low-connectivity settings. This dependency could limit real-time monitoring or access in rural or resource-constrained environments. To address this, future iterations will adopt a hybrid architecture that incorporates edge computing for local signal processing and basic decision support, while maintaining cloud-based storage for long-term data management and visualization. Also, is it important to mention that although no personal identifiers were transmitted in this study, future versions of the system should include secure data transmission protocols and encryption to ensure patient privacy and compliance with healthcare data protection standards.

Fifth, although axis-based spectral filtering helped isolate tremor components, motion artifacts caused by voluntary movements during active tasks (e.g., writing or waving) remain a challenge. These artifacts can confound the signal, particularly when tremor and intentional motion overlap in frequency. Future development will incorporate signal segmentation techniques and machine learning models to distinguish voluntary movement patterns from involuntary tremors in real-time.

Finally, the study did not extensively evaluate the effects of external factors, such as cognitive load, emotional stress, or fatigue, on the severity of tremors. Future research should incorporate these variables to better understand their impact on tremor variability and progression.

Despite these limitations, the ELENA system shows potential as a scalable and objective tool for assessing PD tremors, laying the groundwork for further improvements in wearable and remote monitoring technologies.

### 5.2. Emerging Technologies

The advancement of wearable technology, artificial intelligence, and the connectivity of the IoT has significantly transformed the landscape of remote monitoring systems for Parkinson’s disease. Several emerging technologies and methods can enhance the capabilities of the ELENA system in detecting and analyzing tremor events more effectively.

One key development is the use of ML and DL algorithms to improve tremor classification. By analyzing vast datasets of tremor recordings, ML models can distinguish between different types of tremors—resting, postural, and action tremors—with greater precision. This allows for more personalized tracking and could lead to the earlier detection of disease progression or ineffectiveness of medications.

The implementation of multimodal sensor fusion is another promising approach. By integrating accelerometers, gyroscopes, and EMG sensors, the system can provide a more holistic assessment of motor symptoms. Multimodal sensing improves the accuracy of tremor detection by capturing not only frequency and amplitude variations but also muscle activation patterns, which can help differentiate Parkinson´s tremors from other movement disorders.

The integration of edge computing and embedded AI presents another opportunity for real-time tremor analysis. Instead of relying on cloud-based processing, wearable devices equipped with embedded AI can analyze tremor data locally, reducing latency and ensuring continuous functionality even in environments with poor connectivity. This advancement improves the reliability of real-time monitoring, providing immediate feedback to both patients and healthcare professionals.

The expansion of 5G networks and IoT-based healthcare solutions further enhances the potential of remote monitoring systems such as ELENA. With ultra-low latency and high-speed data transmission, 5G enables continuous, real-time tremor monitoring, facilitating timely clinical interventions and more dynamic treatment plans.

By incorporating these emerging technologies, the ELENA system can evolve into a more sophisticated real-time monitoring solution for Parkinson’s disease.

## 6. Conclusions

The findings of this study highlight the complexity of Parkinson’s disease tremors, demonstrating that the characteristics of tremors vary between different test conditions, age groups, and stages of disease progression. Resting tremors, postural tremors, and action-related tremors exhibit distinct frequency and amplitude patterns, reinforcing the importance of personalized evaluations in clinical practice. Also, the observed increase in the intensity of the tremor with voluntary movement underscores the role of motor and cognitive stress in exacerbating symptoms.

One of the key contributions of this work is the development and evaluation of the ELENA system prototype, a wearable solution designed for real-time tremor monitoring. The system successfully collected tremor data through an IoT-based wearable device and transmitted it to a cloud platform for visualization and analysis. While the current study was conducted in a controlled setting, this architecture demonstrates the potential for future remote monitoring applications. In particular, the platform’s ability to display real-time tremor data and flag unusual fluctuations lays the groundwork for more responsive and personalized care models.

By offering quantitative tremor analysis outside traditional clinical scales, this work supports the future integration of wearable and remote health technologies into the broader PD care ecosystem. However, it is important to note that the current study did not compare tremor data with UPDRS or clinical diagnostic tools, and therefore, no direct clinical conclusions can be drawn at this stage.

Future research will aim to expand the dataset across more diverse patient demographics, increase the duration of monitoring to observe daily variations in tremor behavior, and explore the integration of activity recognition and machine learning to better contextualize and interpret tremor patterns. These enhancements are expected to improve the system’s clinical relevance and support the long-term goal of contributing to better-informed PD management strategies. 

## Figures and Tables

**Table 1:** PD demographics.

Patient	Age	Gender	Years Diagnosed	Medication	Dominant Hand
1	65	Male	5	Levodopa carbidopa ½ tab, Pramipexol 1 mg ½ tab	Right
2	75	Male	8	Levodopa carbidopa ½ tab, Pramipexol 1 mg ½ tab	Right
3	60	Male	4	None	Right
4	73	Female	7	Levodopa carbidopa	Right
5	68	Female	10	Levodopa carbidopa ¼ tab	Right
6	68	Female	9	Levodopa, Pramipexol	Right
7	77	Female	12	Levodopa carbidopa	Right
8	62	Male	2	Pramipexol 0.25 mg	Right
9	66	Male	6	Pramipexol 0.25 mg	Right

**Table 2:** Control group demographics.

Control	Age	Gender	Dominant Hand	Medication
1	64	Male	Right	None
2	70	Female	Right	None
3	62	Male	Right	None
4	65	Female	Right	None
5	67	Male	Right	None
6	68	Female	Right	None
7	72	Male	Right	None
8	63	Female	Right	None
9	69	Male	Right	None

**Table 3:** Detailed summary of accuracy validation results for the Prototype ELENA.

Condition	Metric	Prototype ELENA	Apple Watch	Deviation (%)
Static Testing	Frequency Range (Hz)	1–10	3–10 (Detectable)	<0.5
Dynamic Testing	Frequency Range (Hz)	1–12	3–12 (Detectable)	<0.5
Error Analysis	Average Frequency Error (Hz)	0.02	Baseline	-
Error Analysis	Percentage Error (%)	−0.37	Baseline	-

**Table 4:** Detailed summary of results of clinical tests for the Prototype ELENA.

Patient ID	Test ID	Age (Years)	Years Diagnosed	Dominant Freq. X (Hz)	Dominant Freq. Y (Hz)	Dominant Freq. Z (Hz)	Energy Concentration Range (Hz)	Avg. Amplitude Dominant Range (m/s^2^)
1	1	65	5	4.3519	4.3519	4.3519	3.0–5.0	1.6112
1	2	65	5	4.4049	4.3599	4.4049	3.0–5.0	0.7929
1	3	65	5	5.8124	6.0827	7.2317	5.0–7.0	1.3023
1	4	65	5	1.8769	7.2573	1.9394	1.0–3.0	1.0741
1	5	65	5	0.8148	0.5926	0.8148	1.0–3.0	1.2816
1	6	65	5	0.8418	0.9620	0.8418	1.0–3.0	1.9874
2	1	75	8	3.7177	11.2935	3.7177	5.0–7.0	1.1944
2	2	75	8	7.7614	6.1275	3.7582	5.0–7.0	1.2913
2	3	75	8	6.5899	6.1728	8.2583	5.0–7.0	1.7209
2	4	75	8	5.6081	5.6081	5.8710	5.0–7.0	1.7848
2	5	75	8	0.6536	0.6536	2.3693	1.0–3.0	1.4575
2	6	75	8	NaN	NaN	NaN	NaN	NaN
3	1	60	4	4.3062	2.8506	9.9466	5.0–7.0	0.8045
3	2	60	4	7.9697	7.5828	8.8982	7.0–9.0	1.1463
3	3	60	4	7.0535	4.2176	7.0535	7.0–9.0	0.9343
3	4	60	4	NaN	NaN	NaN	NaN	NaN
3	5	60	4	NaN	NaN	NaN	NaN	NaN
3	6	60	4	0.6270	3.5114	7.3990	3.0–5.0	0.9048
4	1	73	7	3.9683	3.9683	4.0431	3.0–5.0	1.1422
4	2	73	7	4.2301	5.4992	1.9741	5.0–7.0	1.2175
4	3	73	7	4.1249	8.1976	4.1249	3.0–5.0	1.0849
4	4	73	7	5.6096	5.6096	4.1227	5.0–7.0	1.0899
4	5	73	7	NaN	NaN	NaN	NaN	NaN
4	6	73	7	0.7944	0.6355	1.4829	1.0–3.0	0.6899
5	1	68	10	9.8692	3.8257	5.5999	7.0 - 9.0	0.8235
5	2	68	10	1.6920	5.6402	6.3922	5.0 - 7.0	1.0413
5	3	68	10	5.6004	8.4379	4.6296	5.0 - 7.0	1.2352
5	4	68	10	2.9380	4.3403	1.9364	1.0 - 3.0	1.1354
5	5	68	10	1.9960	5.4336	2.0515	1.0 - 3.0	1.0606
5	6	68	10	0.6578	0.6072	9.4111	1.0 - 3.0	0.7640
6	1	68	9	4.4767	4.4579	4.4579	3.0–5.0	0.5721
6	2	68	9	1.6920	5.6402	6.3922	5.0–7.0	1.0413
6	3	68	9	5.7319	5.7319	5.7319	5.0–7.0	1.1137
6	4	68	9	NaN	NaN	NaN	NaN	NaN
6	5	68	9	NaN	NaN	NaN	NaN	NaN
6	6	68	9	0.7663	5.3640	0.6705	5.0–7.0	0.9140
7	1	77	12	3.6353	5.3132	7.2707	3.0 - 5.0	1.6510
7	2	77	12	3.8354	4.2422	3.7192	3.0 - 5.0	1.2830
7	3	77	12	5.1743	5.1743	4.4374	3.0 - 5.0	0.5210
7	4	77	12	4.8864	5.2132	4.9642	5.0 - 7.0	0.5980
7	5	77	12	NaN	NaN	NaN	NaN	NaN
7	6	77	12	4.2735	2.1368	4.2735	3.0 - 5.0	8.9833
8	1	62	2	4.3851	4.4657	4.4174	3.0–5.0	0.6785
8	2	62	2	5.2426	4.6135	5.2426	5.0–7.0	0.5549
8	3	62	2	5.0403	5.0563	5.0403	3.0–5.0	0.4965
8	4	62	2	5.2104	5.2104	5.2104	5.0–7.0	0.6632
8	5	62	2	NaN	NaN	NaN	NaN	NaN
8	6	62	2	NaN	NaN	NaN	NaN	NaN
9	1	66	6	5.3168	5.4796	5.5339	5.0–7.0	1.2753
9	2	66	6	6.3301	5.4364	5.4364	5.0–7.0	1.6584
9	3	66	6	5.6926	5.6926	5.7453	5.0–7.0	0.7262
9	4	66	6	6.0171	6.0171	6.0171	5.0–7.0	1.1919
9	5	66	6	2.6953	4.0704	5.7206	5.0–7.0	0.9993
9	6	66	6	1.1111	1.1111	1.0101	1.0–3.0	1.9980

**Table 5:** Summary of tremor intensity across different age groups. The mean tremor amplitude and standard deviation provide insight into both central tendency and variability.

Age Group	Mean Tremor Amplitude (m/s^2^)	Standard Deviation
60–64	0.7729	0.2167
65–69	1.1636	0.3759
70–74	1.0449	0.2055
75–79	2.0487	2.4743

## Data Availability

Data utilized in this work are available from the corresponding author by request.
